# The Role of Protein Denaturation Energetics and Molecular Chaperones in the Aggregation and Mistargeting of Mutants Causing Primary Hyperoxaluria Type I

**DOI:** 10.1371/journal.pone.0071963

**Published:** 2013-08-27

**Authors:** Noel Mesa-Torres, Israel Fabelo-Rosa, Debora Riverol, Cristina Yunta, Armando Albert, Eduardo Salido, Angel L. Pey

**Affiliations:** 1 Department of Physical Chemistry, Faculty of Sciences, University of Granada, Granada, Spain; 2 Centre for Biomedical Research on Rare Diseases, Instituto Tecnologías Biomédicas, University of La Laguna, Tenerife, Spain; 3 Department of Crystallography and Structural Biology, Instituto de Química Física “Rocasolano”, Consejo Superior de Investigaciones Científicas, Madrid, Spain; Russian Academy of Sciences, Institute for Biological Instrumentation, Russian Federation

## Abstract

Primary hyperoxaluria type I (PH1) is a conformational disease which result in the loss of alanine:glyoxylate aminotransferase (AGT) function. The study of AGT has important implications for protein folding and trafficking because PH1 mutants may cause protein aggregation and mitochondrial mistargeting. We herein describe a multidisciplinary study aimed to understand the molecular basis of protein aggregation and mistargeting in PH1 by studying twelve AGT variants. Expression studies in cell cultures reveal strong protein folding defects in PH1 causing mutants leading to enhanced aggregation, and in two cases, mitochondrial mistargeting. Immunoprecipitation studies in a cell-free system reveal that most mutants enhance the interactions with Hsc70 chaperones along their folding process, while in vitro binding experiments show no changes in the interaction of folded AGT dimers with the peroxisomal receptor Pex5p. Thermal denaturation studies by calorimetry support that PH1 causing mutants often kinetically destabilize the folded apo-protein through significant changes in the denaturation free energy barrier, whereas coenzyme binding overcomes this destabilization. Modeling of the mutations on a 1.9 Å crystal structure suggests that PH1 causing mutants perturb locally the native structure. Our work support that a misbalance between denaturation energetics and interactions with chaperones underlie aggregation and mistargeting in PH1, suggesting that native state stabilizers and protein homeostasis modulators are potential drugs to restore the complex and delicate balance of AGT protein homeostasis in PH1.

## Introduction

Primary hyperoxaluria type I (PH1) is an autosomal recessive inborn error of metabolism caused by mutations in the *AGXT* gene, coding for the enzyme alanine-glyoxylate aminotransferase (AGT). AGT catalyzes the transamination of L-alanine to pyruvate and glyoxylate to glycine in the presence of pyridoxal 5′-phosphate (PLP) as cofactor [1]. AGT deficiency causes glyoxylate accumulation that is subsequently oxidized to oxalate, leading to the production of calcium oxalate crystals that result in progressive renal failure, and eventually, a life-threatening systemic build-up of oxalate known as oxalosis [2]. Liver and kidney transplantation is the only curative option to date, but this aggressive treatment poses significant morbidity and mortality [3]. A limited number of patients with specific genotypes have been reported to respond to pharmacological doses of pyridoxine, even though the molecular mechanisms involved in the response are unclear [4], [5], [6], [7]. *AGXT* has two polymorphic variants, the most frequent (“wild-type”; AGT WT) called the *major* allele (haplotype) and a less common polymorphic variant called the *minor* allele (referred to as AGT LM) which appears in 20% of control subjects and 46% of PH1 patients [1]. The minor allele shows two single amino acid substitutions (p.P11L and p.I340M) among other genomic changes. Even though the minor allele does not cause PH1 by itself, it is known to exacerbate the deleterious effects of additional mutations [1], [4], [7]. About 150 mutations in the *AGXT* gene have been described in PH1 patients, 50% of them being missense mutations [1], and a few of them, such as p.G170R and p.I244T, are relatively common. Several molecular mechanisms seem to contribute to AGT loss-of-function in PH1 at the protein level: i) Mitochondrial mistargeting, where the AGT enzyme is imported to mitochondria [2]; ii) Protein aggregation [4], [8], [9]; iii) Accelerated proteasomal degradation [8]; iv) Catalytic defects [4]. However, the molecular details underlying protein mistargeting, aggregation and degradation in PH1 remain unclear. Beyond their interest in PH1, some of these mutations have important implications in cell biology and genetics. AGT mistargeting mutations are unique models to try to understand some of the principles behind enzyme compartmentalization in the cell, which also has relevant evolutionary connotations [10]. In addition, the necessary synergy between common polymorphisms and disease-causing mutations of the *AGXT* minor haplotype is one of the best characterized examples of such interaction in human genetics [2].

Human genetic diseases are often caused by alterations in protein homeostasis [11]. The ability of a protein to fold into its native and functional conformation relies on intrinsic physico-chemical properties (thermodynamic stability, folding, unfolding, misfolding and aggregation rates) in the crowded intracellular milieu, as well as in the interaction of different conformations populated along the folding/unfolding process with elements of the protein homeostasis network, an array of pathways involved in the control of protein synthesis, folding, post-translational modification, trafficking, disaggregation and degradation [11], [12], [13], [14]. In the context of protein homeostasis, we have recently suggested a role of the low kinetic stability of the apo-AGT (which shows no coenzyme bound [6]) and enhanced interactions with Hsc70, Hsp90 and Hsp60 chaperones [6], [9], [15] in the aggregation (for the I244T variant -p.P11L, p.I244T and p.I340M in *cis* -) and mistargeting (for the G170R variant -p.P11L, p.G170R and p.I340M in *cis* -) of PH1 mutations. Whether these aberrant protein features are specific to these mutations and/or intrinsic to a certain pathogenic mechanism (aggregation vs. mistargeting) remains unclear. From the perspective of the protein homeostasis network, understanding the role of specific protein features in the disease-causing mechanisms is required to develop new therapeutic strategies aimed to restore protein function [11], [13], [14]. However, owing to the large complexity and interactivity of the protein homeostasis pathways, involving at least 800 different proteins [13], a detailed characterization of protein homeostasis defects in PH1 represents a remarkable challenge.

In this work, we have performed a multidisciplinary characterization of four polymorphic variants (WT, p.P11L, p.I340M and p.P11L/I340M or *minor allele*, LM) and six known PH1 mutations present in the *minor allele* (named p.H83R, p.F152I, p.G170R, p.I244T, p.P319L and p.A368T through this paper). We also analyzed two rare variants of uncertain pathogenicity: p.R197Q and p.A295T, both in the *minor allele*. Our main aim is to shed light on the complex mutational effects of AGT folding and stability in PH1 from biochemical, biophysical, cell and structural biology perspectives required to deeply understand PH1 as a conformational disease. Our results show that PH1 causing mutations associated with aggregation and mistargeting display common alterations in protein folding, stability and interaction with molecular chaperones. This suggests that the protein homeostasis pathways involved in both mechanisms are shared and, consequently, that the final fate of the mutant proteins is likely determined by their specific regulatory elements. Nevertheless, our results indicate that native state stability and molecular chaperones are key points to understand PH1 pathogenesis, that might be targeted pharmacologically to restore protein homeostasis in PH1 patients.

## Materials and Methods

### Construction, expression and purification of Pex5p-pbd and AGT proteins in *E.coli*

Cloning of the PTS1-binding domain (amino acids 235–602 in reference sequence NM_000319) of human Pex5 (Pex5p-pbd) was performed after reverse transcription of normal human liver mRNA and PCR amplification with primers BgNPEX5-F: AGATCTCATATGGAGTTTGAACGAGCCAAG and SlRPEX5-R: TGCGACGAATTCACTGGGGCAGGCCAAAC. The NdeI and EcoRI sites designed at the 5′end of the primers were used to clone the amplification product into pCOLDII expression vector. The AGT expression constructs were generated as previously described [6], using site-directed mutagenesis, standard subcloning procedures and confirmed by sequencing. *E. coli* BL21strain containing pCOLDII plasmids encoding AGT and Pex5p-pbd proteins were grown in the presence of ampicillin 0.1 mg/ml and induced with 0.4 mM IPTG for 6 h at 4°C. His-tagged AGT and Pex5-pbd proteins were purified from soluble extracts using IMAC-columns (GE Healthcare) as recommended by the manufacturer. Proteins were further purified by size-exclusion chromatography (SEC) as previously described [6]. Holo- and apo-AGT were prepared and stored as previously described [6]. Protein concentration was measured spectrophotometrically using ε_280(1 mg/ml)_ = 1.069 (AGT) and 1.243 (Pex5p-pbd), calculated from their sequences [16].

### Spectroscopic analyses

All spectroscopic analyses were performed in 20 mM Na-Hepes, 200 mM NaCl pH 7.4 at 25°C. The hydrodynamic behavior of dimeric holo-AGT proteins was evaluated by dynamic light scattering (DLS) using 5 µM AGT (in subunit) and 50 µM PLP in a Zetasizer Nano ZS (Malvern Inc.). UV-visible absorption spectra were acquired in an Agilent 8453 diode-array spectrophotometer using 3 mm path length cuvettes and 20 µM AGT. Near-UV/Visible circular dichroism measurements were performed as described [6]. Fluorescence measurements were performed as previously described [6], [17] with some minor modifications (see SI text).

### Enzyme kinetic analysis

The AGT overall transaminase activity was customarily measured as described [18]. Briefly, 5 µg/ml of AGT incubated in Na-Phosphate 0.1 M pH 8 buffer at 25°C in the presence of 150 µM PLP, 0–5 mM glyoxylate and the reaction was triggered by adding 0–100 mM L-Alanine. Pyruvate formed in the reactions was measured in a Tecan Infinite M200 Pro microplate reader by a coupled NADH:lactate dehydrogenase assay after 2 min reaction at 25°C. Global fittings of activity measurements were performed using a double-displacement mechanism [17].

### Differential scanning calorimetry (DSC)

DSC measurements were performed and analyzed using a two-state irreversible kinetic model as described [6] (a detailed description of DSC fittings can be found in the SI text). Denaturation rate constants *k* are determined from the profiles of excess heat capacity vs. temperature profiles using equation 1:
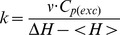
(1)Where *C*_p(exc)_ and <*H*> are the excess heat capacity and the excess enthalpy at each temperature, *ν* and Δ*H* stand for the scan rate and the calorimetric enthalpy, respectively. The temperature dependence of the rate constants follows the Arrhenius equation:
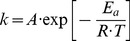
(2)Where *E*_a_ is the activation energy and *T* is the absolute temperature in K.

Mutational effects on the activation free energy (ΔΔ*G* ^‡^), enthalpy (ΔΔ*H* ^‡^) and entropy (ΔΔ*S* ^‡^) where determined on the basis of the transition state theory as described in [19]. The values of ΔΔ*H* ^‡^ and ΔΔ*S* ^‡^ where considered to be constant within the temperature range involved in extrapolations based on the highly linear Arrhenius plots found in all cases as well as the nice agreement between the values obtained upon determination of these parameters at 37°C (physiological temperature) and at 60°C (approximately the average and median *T*_m_ value for all AGT enzymes; data not shown). Mutational effects on activation energetic parameters were determined using equations (3)–(5):
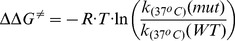
(3)

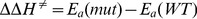
(4)

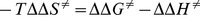
(5)


### Expression and characterization of AGT variants in chinese hamster ovary (CHO) cells

CHO cells (ATTC, USA) were grown in α-MEM (Lonza, Germany) supplemented with glutamine, penicillin/streptomycin and 5% fetal bovine serum. Cell transfections were performed using AGT cDNA variants subcloned in pCIneo plasmids (Promega, USA) in 6-well plates with Transfast reagent (Promega, USA), following manufacturer's guidelines. After 24 h, cells were passed to 100 mm Petri dishes containing 4 glass coverslips for immunofluorescence studies. Cultures were harvested 48 h after transfection.

Cells were sonicated in lysis buffer (100 mM potassium phosphate pH 8.0, 250 mM sucrose, 0.05% triton X-100, 100 µM PLP) and centrifuged at 4°C, 5000 *g* for 10 min (Beckman, USA) to obtain soluble fractions (supernatants). The pellets were washed with phosphate buffer saline (PBS) and resuspended in RIPA buffer. The protein concentration was measured by using bicinchoninic acid (BCA), and equal amounts of total protein were run in 10% acrylamide-SDS gels. The upper portion of the gel (above 70 kDa) was stained with Coomassie blue and scanned to control for equal protein loading, while the remaining of the gel was transferred to nitrocellulose membranes for western analysis. Purified AGT protein were included to calibrate the amount of AGT present in the cell lysates. Membranes were probed with anti-AGT rabbit serum followed by HRP-conjugated anti-rabbit IgG (Jackson ImmunoResearch, USA) and enhanced chemiluminescence substrate (Roche, Germany). Chemiluminescent signals were measured in VersaDoc 4000 MP and ChemiDoc MP devices and analysed using ImageLab Software (BioRad, Hercules, CA).

AGT activities were measured using cell lysates containing 100 µg total protein incubated in 100 mM K-Phosphate buffer pH 8.0 at 37°C for 30 min in the presence of 40 µM PLP, 10 mM glyoxylate and 40 mM L-Alanine. Pyruvate formed in the reactions was measured by a coupled NADH:lactate dehydrogenase assay [18].

For immunofluorescence confocal microscopy, cells grown on two coverslips were pulse-labeled *in vivo* with 100 nM MitoTracker Red (Invitrogen USA) during 15 min, followed by additional 15 min chase in label-free medium. PBS, pH 7.4, was used as a buffer in all subsequent washes and incubations. Cells were fixed in 3% paraformaldehyde at room temperature for 10 min. and permeabilized with 0.1% Triton X100-PBS. For AGT labeling on mitotracker stained cells, rabbit anti-human AGT (a gift from Dr. Danpure, University College London, UK) and Alexa Fluor 488 goat anti-rabbit IgG (Invitrogen, USA) were used. To label AGT and peroxisomes on the same cells, guinea pig anti-human AGT (also provided by Dr. Danpure) and rabbit anti-human PMP70 (Abcam, UK) were used, followed by incubations with Alexa Fluor 488 goat anti-guinea pig IgG (Invitrogen, USA) and Alexa Fluor 555 goat anti-rabbit IgG (invitrogen, USA). The coverslips were mounted with PBS-glycerol. The images were taken with a 60× objective in a confocal laser-scanning microscope (Olympus Inverted IX81, Japan).

### *In vitro* expression of AGT variants in a cell-free system and interaction with Hsc70 chaperones

Cell-free expression of AGT variants was performed in rabbit reticulocyte lysates (TnT system, Promega, USA) at 30°C for 2 h using ^35^S-Met and AGT cDNA variants subcloned in pCIneo plasmids. Protein synthesis was stopped with 100 µg/ml cycloheximide, and 1/25^th^ of the reaction product was set aside for analysis. Hsc70 immunoprecipitation was carried out with the remaining TnT product, using rat-Hsc70 antibodies (Abcam, Cambridge, UK) as previously described [9]. The immunoprecipitated proteins and the initial TnT products were denatured in Laemmli's buffer and analyzed by SDS-PAGE and fluorography.

### Molecular modeling

p.I340M crystallization assays were carried out on a 60-well microbath under oil (Terasaki plates) at 291 K. Crystals were obtained using a precipitant solution containing 15% PEG 3350; 0.1M Bis-Tris pH 5.2, 150 mM Li_2_SO_4_ and 5% w/v octyl-b-D-glucoside as additive. The best crystal forms were obtained by mixing drops of 1 µl protein solution, 2 µl precipitant solution and 0.45 µl additive. In general, the crystals appear and grow in the following 24 hours. Crystals were mounted in a fiber loop, transferred to the cryoprotectant (20% glycerol on the crystal mother liquor solution) and flash-frozen at 100 K in a nitrogen gas steam. p.I340M crystals diffraction data set was collected using a ADSC Q4 CCD detector at ID14.4 beamline of the European Synchrotron Radiation Facility (Grenoble). Diffraction data were processed with XDS [20] and scaled with SCALA from the CCP4 package (Collaborative Computational Project, Number 4) [21]. A summary of the diffraction protocol, data-collection and refinement statistics are given in Table S2. p.I340M diffracted to 1.90 Å resolution and belonged to space group *P*2_1_2_1_2_1_, with unit-cell parameters a = 54.5, b = 103.5, c = 153. The p.I340M structure was solved by molecular replacement using Phaser [22] with the coordinates from the native protein (PDB 1H0C [23]). Several cycles of restrained refinement with PHENIX [24] and iterative model building with COOT [24], [25] yielded to the final model with an R/R_free_ 0.17/0.20. The water structure was also modeled. The stereochemistry of the models was verified with MolProbity [26]. Ribbon figures were produced using PyMol [27]. The coordinates and structure factor amplitudes have been deposited in the Protein Data Bank (PDB code: 2YOB).

### Statistical analysis

For statistical comparison, a two-sample Student's t-test was performed.

## Results

### PH1 causing mutants decrease protein yields and enhance protein aggregation in mammalian cells

**Figure 1:**
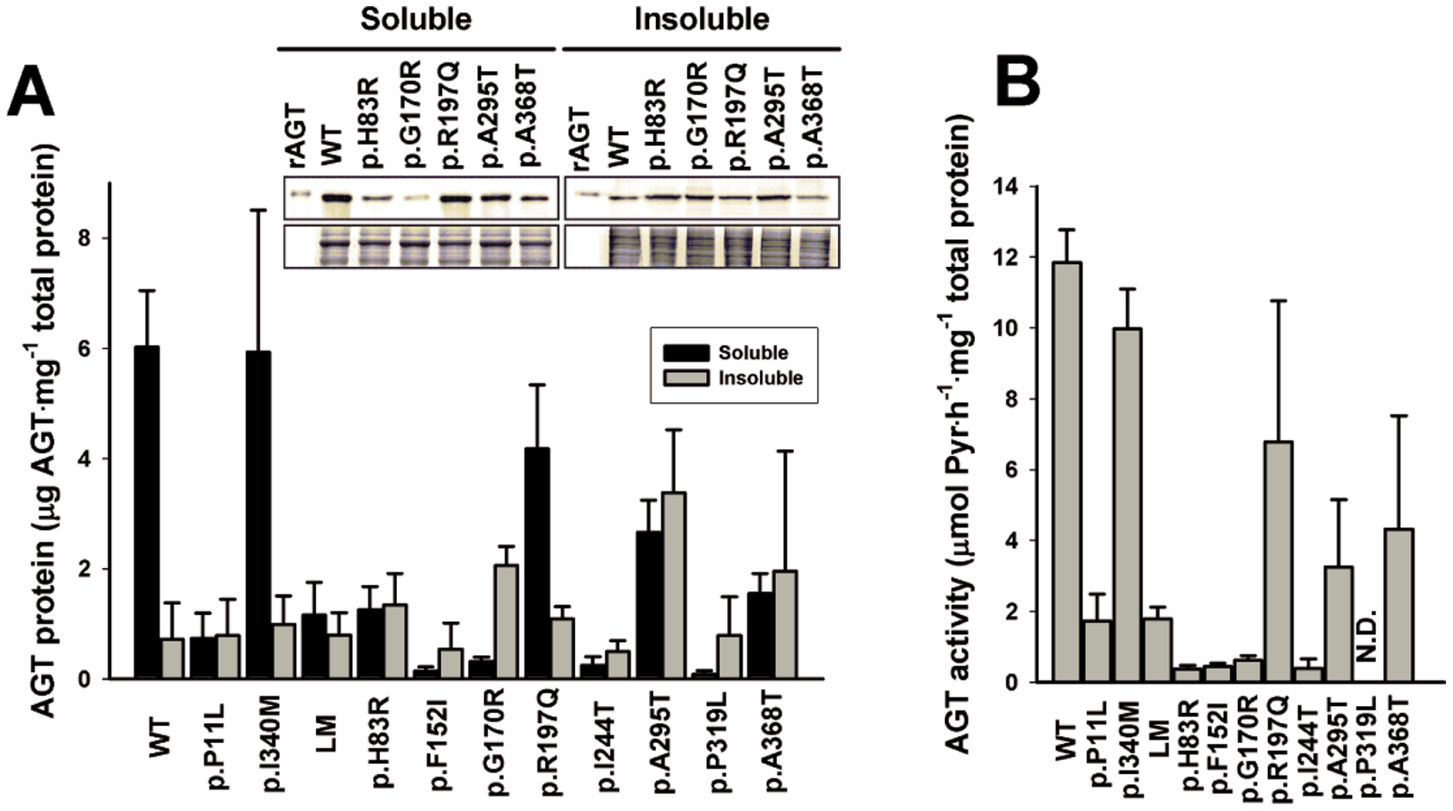
Expression studies of AGT variants in CHO cells. A) Levels of AGT immunoreactive protein as determined in soluble (black bars) and insoluble (grey bars) extracts. The inset shows representative immunodetection experiments of AGT variants (upper image) and the corresponding loading controls (lower image); rAGT: recombinant His-tagged AGT. B) AGT activities in soluble extracts. Data are means±s.d. of 2–5 independent experiments. N.D. not detectable.

**Figure 2:**
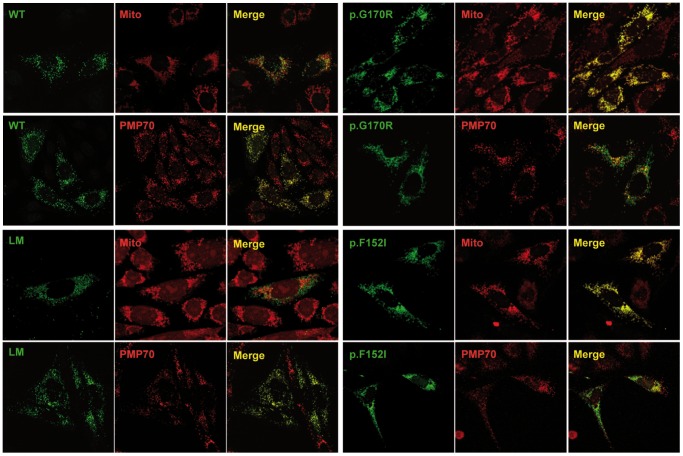
Immunolocalization studies of AGT variants in CHO cells. WT (upper left panel), LM (lower left panel), p.G170R (upper right panel) and p.F152I (lower right panel). In each panel, the upper row shows mitochondrial immunolocalization (AGT variant, Mitotracker probe and their merge), while lower row shows peroxisomal immunolocalization (AGT variant, PMP70 and their merge).

We have explored the impact of mutations and polymorphisms in AGT protein folding efficiency and intracellular trafficking upon transient transfection of CHO cells (Figure 1 and 2). We define AGT intracellular *foldability* as the ability to fold into native dimers inside the cell, which is determined by the partition of the newly synthesized protein into folding, aggregation and degradation pathways. Most of the mutations studied are shown to reduce the total immunoreactive AGT levels (soluble *plus* insoluble in Figure 1A) compared to WT, from ∼8–10-fold (p.F152I, p.P319L and p.I244T) to ∼3–4-fold (LM, p.P11L, p.H83R, p.G170R and p.A368T), while only three mutants show WT-like levels (p.I340M, p.A295T and p.R197Q). Since total AGT protein levels represent the balance between protein synthesis, folding and degradation, these results indicate that most of the PH1 mutants display folding defects, possibly leading to enhanced AGT turnover (remarkably for p.F152I, p.P319L and p.I244T) which would be consistent with previous studies showing increased proteasomal turnover of PH1 mutants in cell-free systems [8], [9].

AGT levels and specific activities (per mg of total protein) in soluble extracts closely correlate (Figure 1). The specific activity (per mg of AGT) measured in these soluble extracts (0.9–1.5 mmol Pyruvate·h^−1^·mg^−1^, with the exception of p.H83R) and in purified AGT dimers (Table 1) are also found to be similar. Therefore, the AGT levels detected in CHO soluble extracts mostly reflect folded AGT dimers. AGT WT exists mainly in the soluble fraction (∼90% of the total AGT protein), while the mutations decrease the presence of AGT in soluble extracts by ∼2-fold (p.P11L and LM, p<0.01; p.A295T, p = 0.054; p.H83R and p.A368T, p = 0.10), ∼3–4-fold (p.F152I and p.I244T, p≤0.01) and ∼8-fold (p.P319L and p.G170R, p<0.01)(Figure 1A). Noteworthy, p.F152I, p.G170R, p.I244T and pP319L showed significantly decreased fraction of AGT protein (p<0.05 in all cases) and specific activity (p<0.01 except for p.P319L which was not detectable) in soluble extracts compared to AGT LM. Overall, these results indicate that p.F152I, p.G170R, p.I244T and p.P319L further reduce AGT intracellular foldability compared to AGT LM also by enhancing protein aggregation.

**Table 1:** Functional properties and hydrodynamic diameter of AGT variants.

AGT Variant	*V*_max_ (mmol·h^−1^·mg^−1^)^a^	*K*_M(Alanine)_ (mM)^a^	*K*_M(Glyoxilate)_ (µM)^a^	*K*_d(PLP)_ (nM)^b^	*k*_on(PLP)_ (M^−1^·s^−1^)	*k*_off(PLP)_ (10^5^) (s^−1^)	Diameter (mean±s.d.; in nm)^c^
WT	2.22±0.09	19.5±1.4	245±29	100±30 (1400±800)	197±33	28±18	8.1±2.3
p.P11L	2.43±0.08	18.3±1.2	185±22	(1070±90)	150±1	16±1	9.8±2.8
p.I340M	3.03±0.10	22.3±1.3	277±27	172±42	N.det.	N.det.	8.2±0.1
LM	2.26±0.09	19.8±1.5	197±27	157±15	N.det.	N.det.	7.8±0.1
p.H83R	0.141±0.005	42.4±2.4	156±19	(1180±380)	1470±160	174±53	8.9±2.3
p.F152I	1.90±0.19	15.7±3.1	201±68	(490±300)	2990±70	146±90	8.9±0.2
p.G170R	2.54±0.13	21.9±2.0	293±42	(1380±170)	125±2	17±2	6.6±0.9
p.R197Q	1.84±0.06	18.1±1.2	168±21	156±34	N.det.	N.det.	9.9±0.7
p.I244T	2.82±0.20	16.8±2.4	193±49	(3060±240)	51±1	16±1	7.1±0.7
p.A295T	2.10±0.10	18.5±1.3	292±30	71±16	N.det.	N.det.	9.5±0.4
p.P319L	1.54±0.13	16.2±2.7	212±60	(240±360)	268±7	6±10	7.3±1.0
p.A368T	2.12±0.13	23.0±2.5	297±50	134±26	N.det.	N.det.	8.2±0.2

a Enzyme kinetic parameters were obtained from global fittings of 2–4 independent experimental series using a double-displacement mechanism;

b *K*_d_ values were estimated from single titrations except for AGT WT protein where *K*_d_ value is the mean±s.d. from three independent titrations. Data in parenthesis are *K*_d_ estimates obtained from the kinetic binding experiments (*K*_d_ = *k*_off_/*k*_on_).

c Hydrodynamic diameter of holo-AGT variants determined by dynamic light scattering (DLS). Data are mean±s.d. of 3–9 independent measurements.

The intracellular targeting of the AGT variants has been also analyzed in CHO cells by immunofluorescence confocal microscopy (Figure 2). Only two mutants were found to mistarget to mitochondria (p.G170R and p.F152I), where AGT is thought to be unable to detoxify glyoxylate [2], while the remaining variants were found at the correct peroxisomal location. Since peroxisomal import of AGT is mediated by the interaction of its peroxisomal targeting sequence (PTS1) with Pex5p [28], a possible explanation for mitochondrial mistargeting would be alterations in the binding of mutant AGT folded dimers with Pex5p. However, calorimetric titrations of native AGT proteins with the PTS1 binding domain of Pex5p (Figure S1) show little or no effects of these PH1 mutations in the molecular recognition by Pex5p (Table S1).

### PH1 causing mutants strongly interact with Hsc70 chaperones

**Figure 3:**
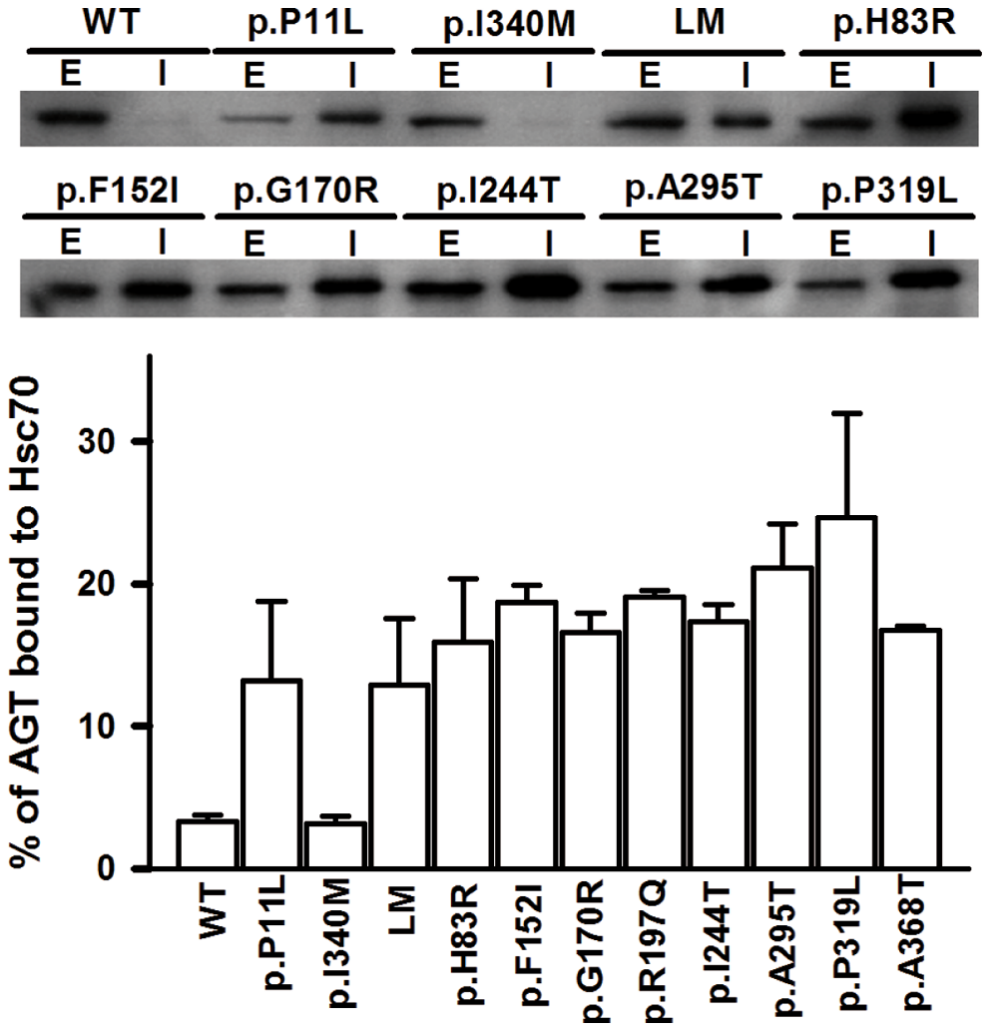
Interaction of AGT variants with Hsc70 chaperones in a cell-free system. Representative autoradiograms of AGT proteins labeled with ^35^S-Met are shown for several AGT variants (E: total AGT synthesized in extracts; I: AGT immunoprecipitated using anti-Hsc70 antibodies; note that 1 µl of the TnT reaction was loaded in E lanes, while the protein immunoprecipitated from 6 µl TnT lysate was loaded in I lanes). Data in the lower panel are expressed as percentage of immunoprecipitated AGT compared to the total AGT synthesized, and are mean±s.d. from three independent experiments.

An alternative explanation for protein misfolding and mistargeting observed for some mutants (see section above) may be that AGT variants abnormally interact with molecular chaperones along their folding process, as previously described by us for the p.G170R and p.I244T variants and chaperones of the Hsp60, Hsp70 and Hsp90 families [6], [9], [15]. Thus, we have tested the interaction of all AGT variants with Hsc70 chaperones upon expression in a cell-free system by immunoprecipitation (Figure 3). All the AGT variants tested showed stronger interactions with Hsc70 chaperones (p<0.01 in all cases), except p.I340M. Polymorphic p.P11L and LM showed a 4-fold increase in immunoprecipitated AGT compared to the WT protein, while disease-causing mutants showed somewhat higher levels, from 4.8-fold (p.H83R, p = 0.46 vs. LM) to 7-fold (p.P319L, p = 0.078 vs. LM). Owing to the significant variability in these experiments, the changes detected are not robust enough to claim mutation-specific differences compared to AGT LM, but the trend observed suggests that disease-causing mutations may increase the interaction with Hsc70 chaperones along their folding process vs. LM protein.

### PH1 causing mutants do not generally perturb AGT oligomerization and function

To probe whether PH1 mutants may affect AGT oligomerization and catalytic properties (as recently found for some PH1 mutants [4]), we have expressed recombinant AGT variants in *E.coli* and purified AGT dimers to homogeneity. Size-exclusion chromatography analysis showed a single peak for all variants with an elution volume consistent with a ≈90 kDa dimeric form (data not shown). The molecular dimensions of purified dimers were further studied by dynamic light scattering (DLS), showing a hydrodynamic diameter of 8.1±2.3 nm for holo-WT and 8.4±1.1 nm for the rest of holo-AGT variants (Table 1). Within the experimental uncertainty, these results are consistent with the size of dimeric AGT WT previously reported by DLS [29] and also imply no noticeable perturbation of dimer size and/or dimer-monomer equilibrium by these mutations.

**Figure 4:**
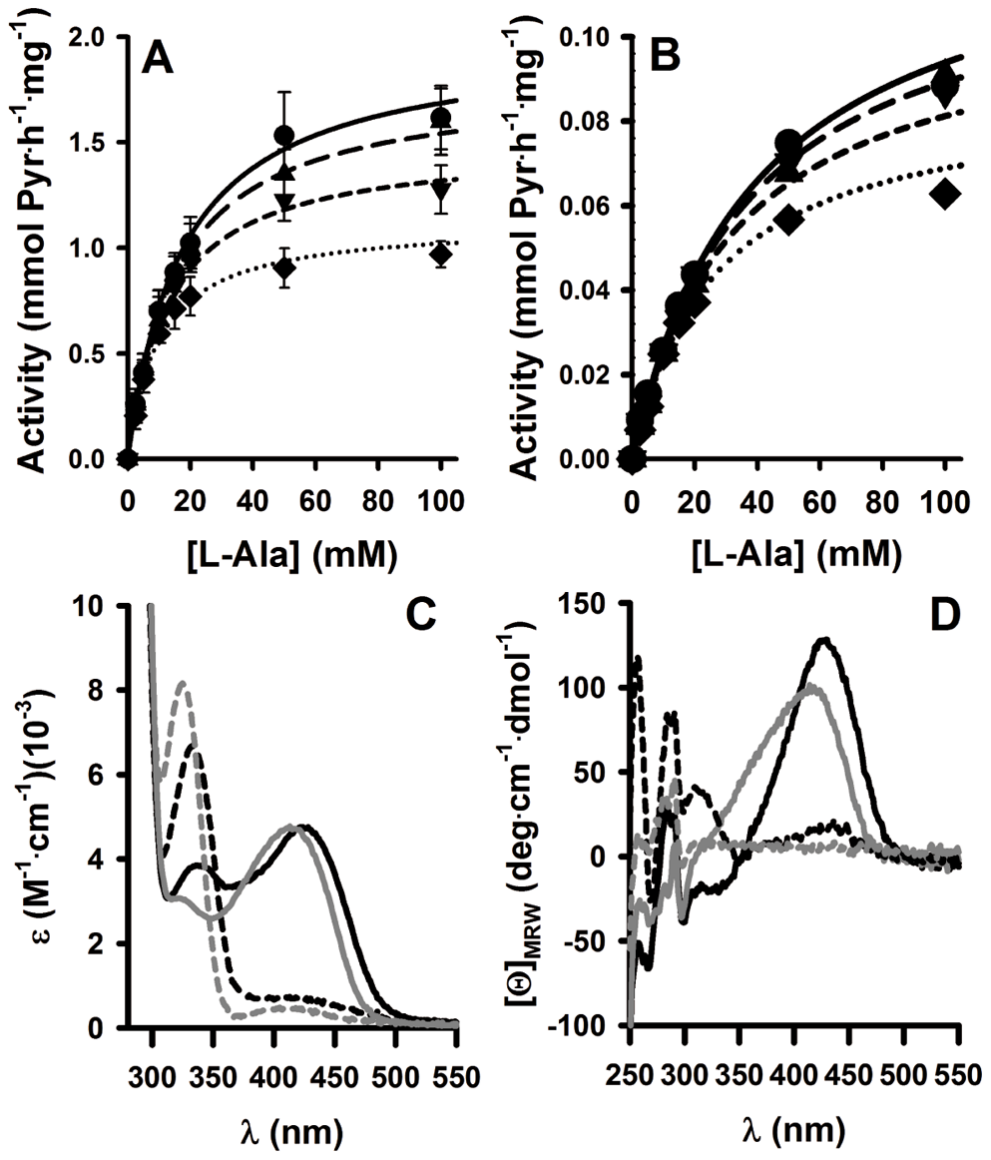
Functional characterization of WT and p.H83R. A and B) Enzyme activity measurements for WT (A) and p.H83R (B) at different L-Ala concentrations in the presence of 0.25 mM (diamonds), 0.5 mM (down triangles), 1 mM (up triangles) and 2 mM (circles) glyoxylate. Data in panel A are means±s.d from four independent measurements while data in panel B are means from two independent measurements. Lines are best fits for the different glyoxylate concentrations obtained from global fittings using a double-displacement mechanism. C and D) Absorption (C) and circular dichroism (D) spectra for holo-WT (black) and holo-p.H83R (grey) acquired upon incubation for at least 10 min in the absence (continuous lines; PLP bound) or presence (dashed lines; PMP bound) of 500 mM L-alanine.

Thus, we evaluated the effect of these variants on the functionality of the AGT protein. Enzyme kinetic analyses based on a double-displacement mechanism ([17]; see Figure 4A–B for representative examples) were performed for all the AGT variants and the enzyme kinetic parameters obtained are compiled in Table 1. All the AGT variants tested showed similar specific activity (*V*_max_) to that found for AGT WT with the exception of p.H83R mutant, which displayed a ∼15-fold reduction in specific activity. No large changes in *K*_m_ values for L-Ala and glyoxylate were found among the AGT variants studied. The environment of the bound coenzymes to WT and p.H83R was characterized by Near-UV and visible absorption and circular dichroism spectroscopies, revealing a ∼10–15 nm blue shift in the absorbance and dichroic bands of bound PLP and PMP (Figure 4C–D), which supports a distorted coenzyme binding mode to the p.H83R mutant. We have further tested this hypothesis by incubating holo-WT and p.H83R (at a final monomer concentration of 36 µM) in the presence of L-Ala for 1 h at 25°C and analyzed the the amount of PMP released by UV-absorption spectroscopy upon filtration using microfilters of 10 kDa cut-off. Under these conditions, ∼3% of the PMP was released in the WT enzyme (consistent with tight binding of PMP along the overall transamination reaction previously reported; [17]), while ∼44% of PMP was released in the p.H83R, indicating a large decrease in PMP binding affinity for this mutant. Thus, we conclude that the large decrease in catalytic performance of p.H83R is caused by a distortion in the binding mode of PLP and PMP coenzymes.

We have also measured the binding affinity of the apo-variants for PLP using fluorescence spectroscopy (Figure S2). Direct equilibrium measurements allowed to determine the *K*_d_ values for the WT and some stable mutants at 30°C (Table 1). All the AGT variants studied under equilibrium conditions showed affinity for PLP similar to the WT protein (*K*_d_ about 100 nM). Under these conditions, aggregation in the absence of PLP was found for p.P11L, p.H83R, p.F152I, p.G170R, p.I244T and p.P319L mutants. Lowering the temperature or adding 10% glycerol did not prevent aggregation in the time scale required for AGT:PLP equilibration. Alternatively, we estimated the affinity for PLP by performing kinetic binding experiments under pseudo-first order conditions (Figure S2B) providing only an upper limit for the *K*_d_ values mostly due to the large uncertainties associated to the determination of the *k*_off_ (Table 1). No clear differences were observed for the estimated *K*_d_ values for PLP between WT and the mutants studied using this kinetic approach (Table 1).

### PH1 mutations kinetically destabilize the apo-AGT form

**Figure 5:**
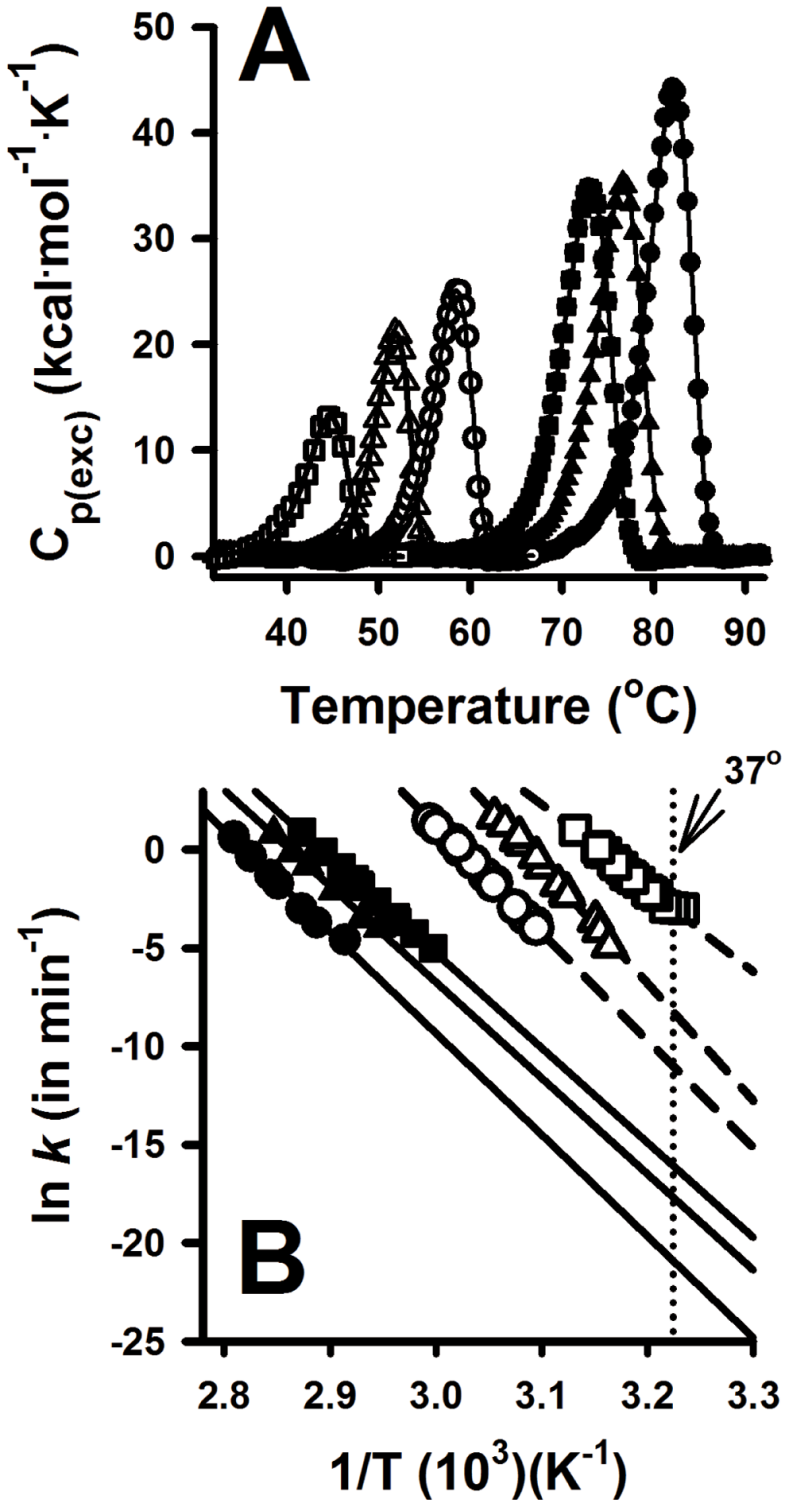
Thermal denaturation of AGT variants studied by differential scanning calorimetry (DSC). A) Representative DSC traces obtained at 3°C/min; Lines are best-fits from a two-state irreversible denaturation model with first-order kinetics [6]; B) Arrhenius plots for the irreversible denaturation of AGT variants, indicating also the extrapolated rate constants at physiological temperature (intercept with the vertical dotted line). Symbols are: WT (circle), LM (triangle) and p.F152I (square). Data for holo proteins are shown as closed symbols and for apo proteins as open symbols.

Thermal denaturation of AGT shows a single transition for all AGT variants studied by differential scanning calorimetry (DSC; Figure 5A). Denaturation of AGT variants is a purely kinetic process, which is well described by the irreversible two-state conversion of the native dimer to a final aggregated state (N→F). This kinetic process is characterized by a denaturation rate constant *k* [6], [30], which is inversely proportional to its half-life for denaturation. In this scenario, protein kinetic stability (as a given denaturation rate or half-life under certain experimental conditions) is determined by the height of the free energy barrier that AGT must cross from the native state to reach the transition state of the denaturation rate-limiting step [7]. Thus, mutational and PLP effects on AGT thermal transition (*T*_m_ and *E*_a_ values) are translated into changes in kinetic stability (Table 2 and Figure S3). Remarkably, removal of bound PLP has a dramatic effect on the stability of most of AGT variants, reducing by ∼25°C and 4–5 orders of magnitude the *T*_m_ values and the corresponding kinetic stabilities (Table 2 and Figure S3).

**Table 2:** Thermal denaturation and kinetic stability parameters for AGT enzymes.

	*T*_m_ (°C)^a^	*K*_37°C_ (min^−1^)^b^^,^ c	Δ*h*_cal_ (kcal·mol^−1^)^b^^,^ d	*E*_a_ (kcal·mol^−1^)^e^	μ^b^
*Holo-AGT*					
WT	82.1	6.4±2.2·10^−10^	548±5	109±5	0.95±0.02
p.P11L	73.8	1.1±0.2·10^−7^	400±4	95±3	0.98±0.01
p.I340M	84.1	1.7±1.1·10^−11^	530±24	115±4	0.90±0.03
LM	76.6	9.3±0.9·10^−9^	375±13	101±5	1.00±0.01
p.H83R	58.2	2.9±0.4·10^−3^	236±15	55±3	0.90±0.03
p.F152I	73.1	9.9±3.3·10^−8^	454±18	97±4	0.92±0.04
p.G170R	75.5	3.7±0.6·10^−8^	458±17	100±2	0.99±0.01
p.R197Q	77.9	3.7±0.7·10^−9^	401±6	110±6	0.96±0.01
p.I244T	75.8	1.1±0.1·10^−8^	405±10	103±2	0.95±0.02
p.A295T	77.5	1.2±0.2·10^−8^	500±9	105±8	0.91±0.01
p.P319L	76.5	8.6±1.5·10^−9^	395±5	101±3	0.98±0.04
p.A368T	76.5	6.8±2.1·10^−9^	440±6	102±7	0.98±0.04
*Apo-AGT*					
WT	58.4	1.6±0.7·10^−5^	255±17	111±10	1.30±0.02
p.P11L	49.9	2.1±0.8·10^−3^	201±14	105±3	1.14±0.01
p.I340M	61.1	2.0±1.0·10^−7^	370±21	143±11	1.04±0.03
LM	51.8	2.6±1.5·10^−4^ (3.7±2.0·10^−4^)	204±10	121±6	1.15±0.06
p.H83R	46.9	3.8±0.8·10^−2^	156±7	62±6	0.91±0.04
p.F152I	44.7	4.9±0.4·10^−2^	137±17	87±7	1.13±0.04
p.G170R	48.5	6.8±0.5·10^−3^ (9.2±3.0·10^−3^)	201±13	92±9	1.00±0.04
p.R197Q	52.3	5.5±0.5·10^−4^	200±4	110±10	0.96±0.06
p.I244T	47.3	3.6±0.4·10^−2^ (2.6±0.5·10^−2^)	162±10	63±1	0.88±0.02
p.A295T	49.4	5.3±1.2·10^−3^ (2.4±0.7·10^−3^)	244±14	92±9	0.89±0.01
p.P319L	47.1	3.9±0.2·10^−2^	194±37	62±2	0.88±0.01
p.A368T	51.8	5.9±0.5·10^−4^	201±3	112±6	1.15±0.06

a determined at 3°C/min scan rate.

b mean±s.d. from independent experiments performed at three different scan rates.

c Values in parentheses are the inactivation rate constants measured at 37°C.

d expressed per mol of AGT dimer (i.e. the unfolding cooperative unit).

e mean±s.d. from the four consistency tests proposed by [47].

**Figure 6:**
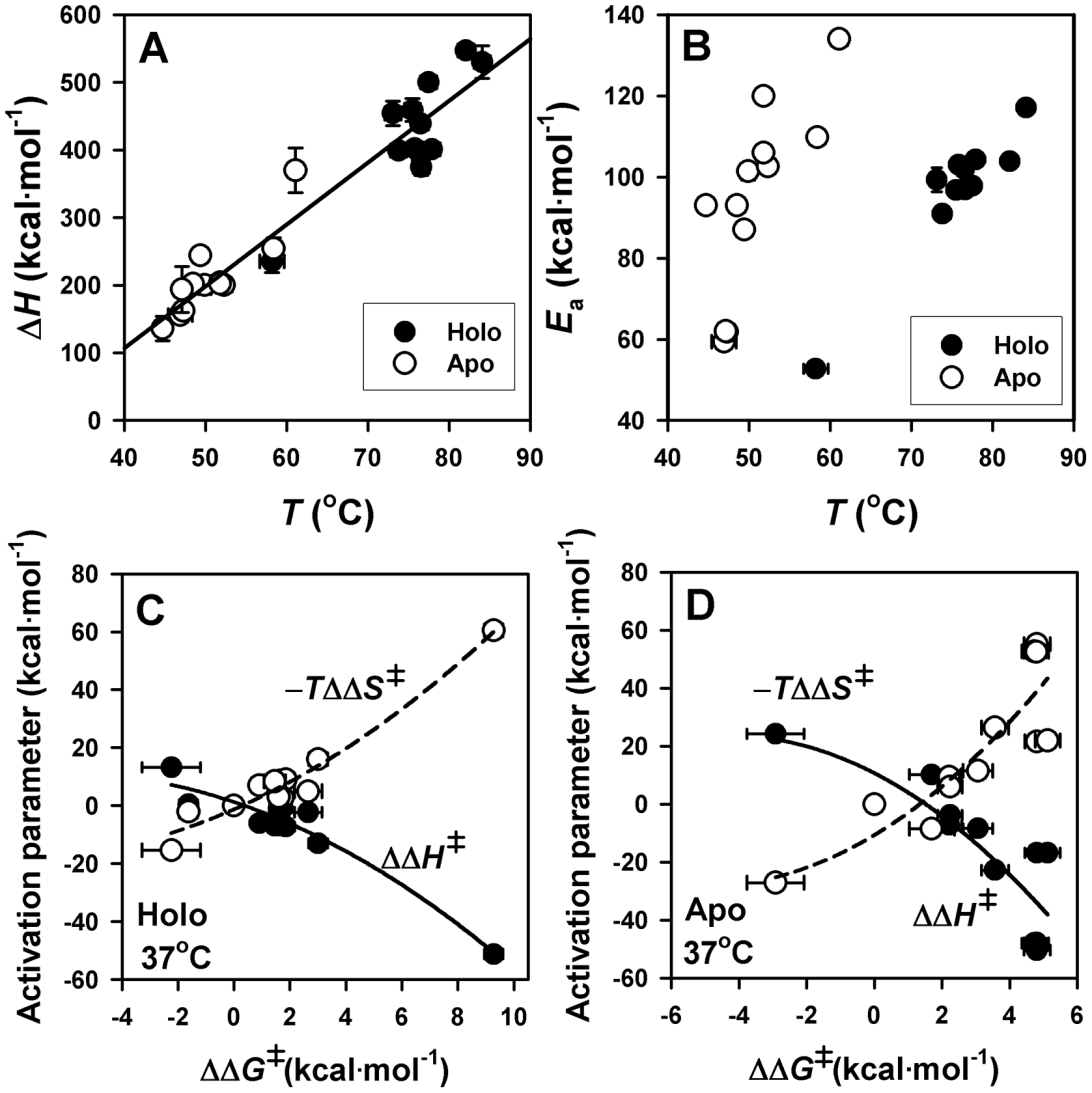
Structure-energetics relationships for thermal denaturion of holo- and apo-AGT enzymes. A) Temperature dependence of denaturation enthalpies (Δ*H*) for holo- (closed symbols) and apo-(open symbols) proteins. The linear fit provides the value of ΔC_p_ ( = 10.2±0.6 kcal.mol^−1^). B) Activation energy (*E*_a_) plotted vs. the *T*_m_ for holo- (closed symbols) and apo-(open symbols) AGT enzymes. C and D) changes in activation enthalpic and entropic contributions to AGT kinetic stability as a function of changes in activation free energies for holo-(C) and apo-(D) AGT enzymes. Lines in C and D are meant to guide the eye and have no theoretical meaning.

DSC is particularly suitable to study the stability of AGT enzymes because it provides not only a *T*_m_ value, which can be alternatively obtained by other techniques, but also allow determine accurately the rate constants as a function of temperature, to compare kinetic stabilities over widely different time scales (in this work half-lives for denaturation range from several minutes to thousands of years, extrapolated to physiological temperature; Table 2) and provide insightful parameters of the denaturation process such as denaturation enthalpies and activation energies [6], [19], [30]. For instance, the temperature dependence of denaturation enthalpies shows a common behavior for holo- and apo-AGT enzymes (Figure 6A), yielding a global slope (i.e. a denaturation heat capacity) of 10.2±0.6 kcal·mol^−1^·K^−1^ which is slightly lower than the theoretical value expected for global unfolding of the AGT dimer (14.8 kcal·mol^−1^·K^−1^; based on [31]). This analysis supports that AGT thermal denaturation involves a large loss of tertiary structure, possibly reflecting denaturation of both domains in AGT, and also that all the AGT enzymes studied here in their holo- and apo-forms denature to a similar extent.

DSC scans are used to determine denaturation rates (*k*) or half-lives at different temperatures by building Arrhenius plots (Figure 5B) which allow study kinetic stabilities at physiological temperature (Table 2). Most of the holo variants are kinetically stable at 37°C with extrapolated half-lives ranging from ∼12 to ∼80000 years (Table 2), with the exception of p.H83R, with a very short half-life of ∼4 h likely due to a distortion in the binding mode of PLP (Figure 4). The apo-proteins are much less stable, remarkably the p.G170R, p.A295T, p.H83R, p.F152I, p.I244T and p.P319L enzymes, which reduce ∼20–150-fold the kinetic stability vs. the LM variant, and they denature on a time scale of a few minutes to hours (Table 2). Indeed, the half-lives for enzyme inactivation upon incubation of apo-proteins at 37°C and standard activity measurements agree very well with those obtained from our DSC analyses (Table 2). This significant kinetic destabilization of the apo vs. holo-forms in most of PH1 mutants is not due to large changes in the affinity for PLP (Table 1), but rather, it suggests that cofactor binding overcomes some destabilizing interactions present in the native state of the apo-forms [7]. Regarding the polymorphic variants, p.P11L and LM are kinetically destabilizing, while p.I340M enhances AGT kinetic stability compared to the WT protein, either in the apo- or the holo-form (Table 2).

The large kinetic stabilization induced by PLP binding, as well as the destabilization induced by some mutants in their apo-form must originate from changes in the height of the denaturation free energy barrier (i.e. the free energy difference between the native and denaturation transition state [6]). Our DSC analyses also provide information on the reaction order of AGT denaturation by determining the μ value (i.e. reaction order is 1/μ), and hence, on the oligomerization state of the denaturation transition state: a μ value close to one indicates first-order kinetics, involving a dimeric transition state for AGT, while a value close to two supports that the transition state is monomeric. As we show in Table 2, most of the AGT variants display first-order denaturation kinetics (μ≈1), with only small deviations for a few apo-forms. Thus, the AGT denaturation transition state is essentially a partially unfolded dimer for all AGT variants, indicating that other unfolding steps (dimer dissociation and monomer unfolding) must occur after the denaturation rate-limiting step, and hence, these steps do not contribute to the effect of mutations, polymorphisms or PLP binding on AGT kinetic stability. PLP mediated kinetic stabilization arise from its preferential binding and stabilization of the native AGT dimeric structure, raising the denaturation free energy barrier by ∼7 kcal·mol^−1^ (see [6] and Table 1).

### Structural and energetic basis of AGT kinetic destabilization by PH1 mutations

A plot of the activation energies for denaturation of all AGT enzymes as a function of their *T*_m_ value (which exponentially correlate well with their kinetic stabilities; see Figure S3) shows that those AGT enzymes with lower kinetic stability display lower values of *E*_a_ (Figure 6B). The high linearity of Arrhenius plots (Figure 5B) indicates a negligible activation heat capacity, and thus, *E*_a_ values can be considered essentially as temperature-independent. Hence, the results shown in Figure 6B can be rationalized as mutational effects on the *energetic balance* (entropic and enthalpic contributions) of the free energy barrier for denaturation (previously shown for denaturation of other protein systems; [19], [32], [33]). We have thus calculated the changes in the activation enthalpies (ΔΔ*H* ^‡^) and entropies (−*T*ΔΔ*S* ^‡^) and plotted them as a function of the mutational effects on the kinetic stability (ΔΔ*G* ^‡^) for the holo-(Figure 6C) and apo-(Figure 6D) enzymes. We found that changes in kinetic stability (ΔΔ*G* ^‡^) arise from large changes of opposite sign in ΔΔ*H* ^‡^ and −ΔΔT*S* ^‡^ that largely cancel out for holo- and apo-AGT enzymes. Since ΔΔ*H* ^‡^ reflect structural differences (∼solvent exposure) between the native and the transition states, the analyses shown in Figure 6 support that the most destabilizing mutants decrease the magnitude of the structural change occurring between the native and denaturation transition states. We must note that there is no experimental evidence of large structural changes in the native dimer upon mutation: they show similar hydrodynamic radius and, with the exception of p.H83R, similar enzyme activities (Table 1) as well as denaturation enthalpies consistent with similarly folded native states (Table 2), and thus, these changes may primarily affect the denaturation transition state. Using structure-energetics relationships, a value of −60 kcal⋅mol^−1^ for ΔΔ*H* ^‡^ (found for several AGT mutants; see Figure 6) is translated into a difference of ∼90 folded residues or ∼8500 Å^2^ of solvent exposed surface [31] between the native and transition state (per dimer), supporting the existence of significant mutational effects on the structure and energetics of the denaturation transition state (see [33] for a similar situation with mutants of human phosphoglycerate kinase 1).

### Structure of p.I340M and modeling of the AGT variants

**Figure 7:**
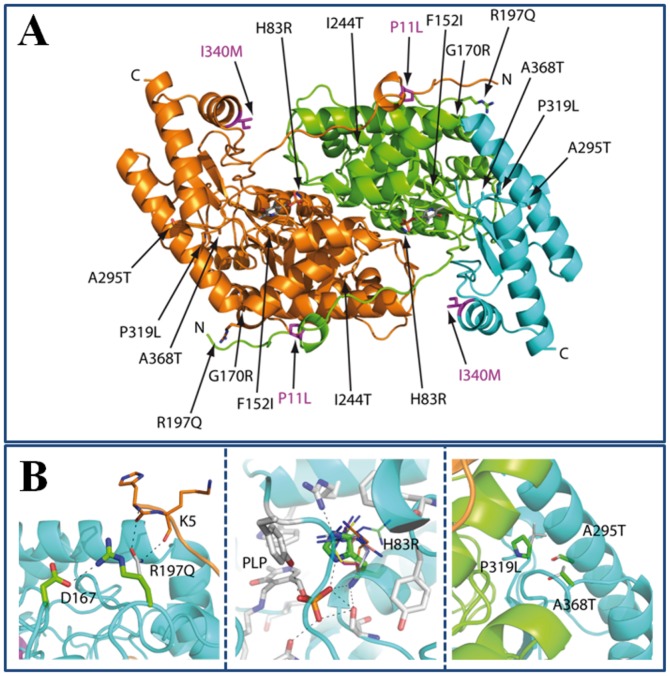
Structural modeling of AGT mutations and polymorphisms. A) Representation of AGT dimer structure. The domain structure of one of the subunits is emphasized using green and blue colors. The AGT mutations found in PH1 patients are labeled and pointed by the arrows while the polymorphisms constituting the *minor allele* are highlighted in magenta. B) Representation of the structural environment of the Arg197 (left), His83 (middle) and Ala295, Pro319 and Ala368 (right). The mutations have been modeled as thinner sticks.

The crystal structure of p.I340M has been determined at an unprecedented 1.9 Å resolution to improve our modeling efforts on PH1 causing mutants. The structures of p.I340M and the WT (PDB code 1H0C [23]) are nearly identical. AGT homodimer has each protomer folded into a large N-terminal domain, a smaller C-terminal domain and a 22 amino acid long unstructured N-terminal tail that grabs the other subunit within the dimer (Figure 7A). None of the substitutions observed in the same haplotype are clustered, and thus, the effects of these substitutions are likely to be additive rather than synergic. The P11L and I340M polymorphisms are found in the dimer interface. P11L is expected to produce steric clashes with residues from the other subunit, which would explain the reduced stability and foldability of p.P11L, while I340M establishes several new favorable interactions hence explaining the stabilization observed for p.I340M, and the partial compensation of P11L destabilizing effect when they both occur in *cis* (i.e. the *minor* allele).

The mutations analyzed on the minor allele occur in locations far from the dimerization interface (Figure 7A), and with the exception of H83R (Figure 7B), none of them are found near the active site. Modeling of Arg83 predicts a loss of interactions with PLP and the appearance of repulsions with surrounding residues, likely affecting the orientation of PLP (and/or PMP), which could explain the low catalytic activity, altered PLP/PMP binding mode and low stability as holo-protein of H83R. A295T, P319L and A368T substitutions are found in the vicinity of the domain:domain interface of AGT monomer (Figure 7B), and cause an increase in the size of the side chain. Interestingly, these three mutations show more pronounced apo-AGT destabilization (Table 2) and deleterious effects in CHO cells (as total and soluble AGT protein levels; Figure 1) as closer the substitution is to the domain interface (P319L>A295T≥A368T), suggesting that a fine tuning of interactions in AGT domain:domain interface may be important for apo AGT kinetic stability and intracellular stability and foldability. G170R, F152I and R197Q mutations are not expected to cause important alterations on AGT structure based on the simple modeling of these substitutions or the crystal structures available [34], although molecular dynamic simulations have suggested a significant structural impact of the F152I mutation [35].

## Discussion

**Figure 8:**
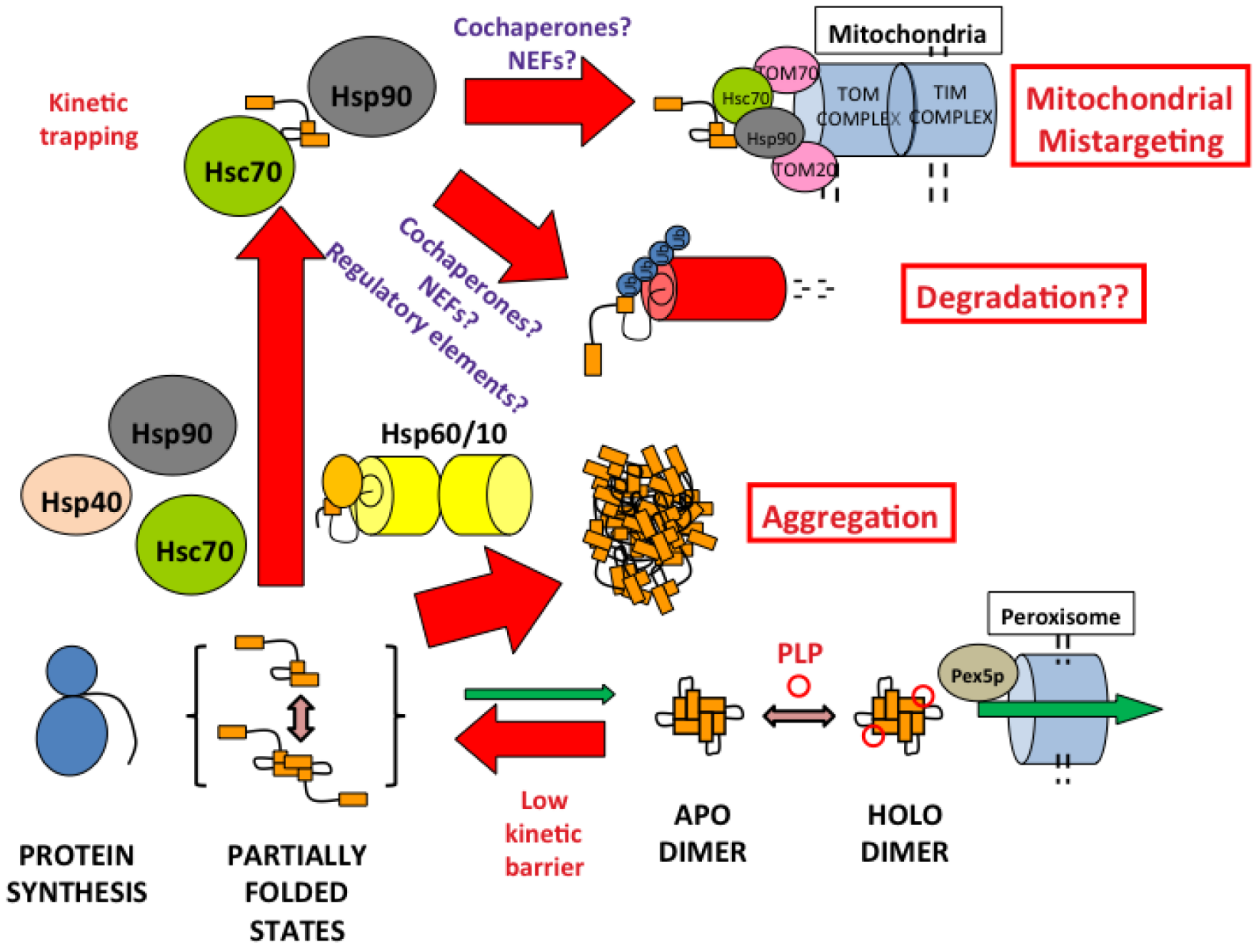
Folding and misfolding checkpoints of PH1 causing mutants.

A majority of the most common alleles associated to PH1 affect protein folding and stability, suggesting that most PH1 patients may show defective AGT function due to alterations in its protein homeostasis (this work; [4], [6], [9], [36]). To understand the different mechanisms underlying PH1, we have integrated in this work concepts from protein structure, biophysics, biochemistry and cell biology to provide a comprehensive view on the mutational effects on protein folding, assembly, transport, mistargeting and degradation of the AGT protein (summarized in Table 3). Our work delineates important checkpoints in AGT protein homeostasis, such as the stability of the apo-proteins and the recognition of folding intermediates by molecular chaperones (Figure 8) that might be specifically targeted to restore AGT function in PH1 patients. We also provide insight to current potential therapies for PH1 such as pyridoxine supplementation [4], [5], [6], [37].

**Table 3:** Summary of mutational effects on molecular properties of AGT protein.

AGT enzyme	Kinetic stability		Eukaryotic cell expression				Interaction with Hsc70	Structural effect
	Holo	Apo	Total AGT protein	Soluble AGT protein	AGT activity	Targeting		
WT	↑	↑	↑↑	↑	↑	Peroxisomal	↓↓	
p.P11L	↓	↓	=	=	=	Peroxisomal	=	Destabilizing clashes between protomers (this work)
p.I340M	↑↑	↑↑	↑↑	↑	↑	Peroxisomal	↓↓	New stabilizing interactions with Q23 and D52 (this work)
LM	=	=	=	=	=	Peroxisomal	=	Combination of P11L and I340M (this work)
p.H83R	↓↓↓	↓↓	=	=	↓↓	Peroxisomal	= ↑	Structural rearrangement at the active site and destabilization of bound cofactors (Figure 7B; this work)
p.F152I	↓	↓↓	↓	↓	↓	Mitochondrial	= ↑	Conservative change (this work); Creates a cavity in the active site displacing W108 which stacks PMP [35]
p.G170R	=	↓↓	=	↓↓	↓↓	Mitochondrial	= ↑	Local conformational rearrangement [34]
p.R197Q	↑	=	↑↑	↑	↑	Peroxisomal	= ↑	Disruption of a salt bridge (R197-D167) and formation of a new hydrogen bond (Q197-K5; Figure 7B; this work)
p.I244T	=	↓↓	↓	↓	↓	Peroxisomal	= ↑	α-helix destabilization by a new hydrogen bond [23]
p.A295T	=	↓↓	↑	=	=	Peroxisomal	↑	Destabilization of domain:domain interface by a bulkier side-chain (Figure 7B).
p.P319L	=	↓↓	↓	↓↓	↓↓	Peroxisomal	↑	Destabilization of domain:domain interface by a bulkier side-chain (Figure 7B). P319 is at the interface.
p.A368T	=	=	=	=	=	Peroxisomal	= ↑	Destabilization of domain:domain interface by a bulkier side-chain (Figure 7B).

Molecular properties (kinetic stability, expression analyses in CHO cells and interaction with Hsc70 chaperones) are semiquantitatively compared taking AGT LM as reference.

The symbols indicate: ↑/↓, increased/decreased;  = ↑, slightly increased;  = , unchanged.

PLP binding to AGT enhances native state kinetic stability by 4–5 orders of magnitude ([6]; this work), making holo-proteins highly kinetically stable at physiological temperature, with the only exception of the catalytic mutant p.H83R. Interestingly, we found that p.F152I, p.G170R, p.I244T, p.A295T and p.P319L are markedly destabilized as apo-proteins compared to the non-pathogenic LM variant (Table 2), and actually, they denature at a relatively fast rate at physiological temperature. As we have previously discussed for p.G170R [6], the low kinetic stability of these mutants in the apo-form may have important implications for PH1 pathogenesis, since it is likely that a significant fraction of AGT may transiently exist as apo-protein *in vivo*, and thus, it might be susceptible to intracellular irreversible alterations such as mitochondrial import, protein aggregation and degradation. Consequently, four of these variants (p.F152I, p.G170R, p.I244T and p.P319L) show evident signs of misfolding and, possibly, accelerated turnover in CHO cells, while two of them (p.G170R and p.F152I) also cause protein mitochondrial mistargeting (Table 3). This indicates some degree of correlation between the apo- stability and protein mitochondrial import and/or intracellular aggregation. According to this interpretation, we propose a beneficial effect of pyridoxine supplementation in patients carrying these four mutations (F152I, G170R, I244T and P319L). In fact, p.F152I and p.G170R have been described as pyridoxine-responsive genotypes in PH1 patients [38], [39].

Our DSC analyses provide molecular insights on the effect of PH1 causing mutants and PLP binding on the AGT kinetic stability, denaturation mechanism and structural/energetic features of its denaturation free energy barrier previously unexplored. Within the set of PH1 mutants studied here, only the thermal stability of p.F152I and p.G170R have been reported earlier (by circular dichroism and inactivation experiments) showing thermal destabilization (lower *T*_m_) mainly for their apo-forms [35], [40], [41]. Our detailed DSC kinetic analyses further show that the kinetic overstabilization exerted by PLP bound to p.F152I, p.G170R, p.I244T, p.A295T and p.P319L as holo-proteins arises from subtle changes in the enthalpic and entropic contributions to the denaturation free energy barrier, since a similar pattern of enthalpy/entropy compensation is found for holo- and apo-AGT proteins (Figure 6C and 6D). Moreover, these effects must concern mainly to the structure and energetics of the dimeric transition state for denaturation, and thus, mutational effects on dimer dissociation and full monomer unfolding do not contribute to the relevant kinetic stability of AGT enzymes since they occur after the denaturation rate-limiting step.

The intracellular homeostasis of AGT protein seems to rely on a delicate balance between protein folding, misfolding, degradation and intracellular trafficking. Importantly, the AGT LM protein, a variant which is not disease-causing but it is known to sensitize AGT towards deleterious mutations, is shown to notably reduce protein kinetic stability and to enhance protein misfolding and degradation, while most of disease-causing mutations further exacerbate at least some of these defects (Table 3). The partial correlation between these molecular defects suggests that multiple elements in the protein homeostasis networks play a role in determining the fate of PH1 mutants (including chaperones, cochaperones and regulatory proteins [13] and vitamin B6 salvage enzymes implicated in the recycling and targeting of PLP to apo-enzymes [42]). This complexity in the homeostasis of AGT proteins, as well as individual differences in the protein homeostasis network (which may even occur among isogenic individuals [43]), may explain inter-individual variability in clinical presentations and residual activities for patients sharing a given genotype [7], [44] and the different fate of mutant proteins (aggregation vs. mitochondrial mistargeting) when expressed under different experimental conditions (this work and [36]). Despite our findings, those specific events and interactions responsible for the partition between protein mitochondrial mistargeting, aggregation and degradation remain elusive.

In the present work, we show that all PH1 mutations of the *minor* haplotype strongly interact with Hsc70 chaperones, adding to our previous work on p.I244T and p.G170R that also showed enhanced interactions with Hsc90 [6], [9] and bacterial Hsp60 [15]. We have recently reported that p.G170R interacts with Hsc70 and Hsp90 chaperones through a *molten globule* folding intermediate [6], while p.I244T interacts with Hsp60 chaperones in partially folded monomeric state with the folded N-terminal and C-terminal domains in an *open* conformation [15]. We thus propose that the last steps involving docking of tertiary structure elements and acquisition of the dimeric quaternary structure are crucial for proper folding of AGT. Moreover, enhanced interaction of PH1 mutants with these molecular chaperones suggest a rougher folding landscape for these mutants (with a higher population of kinetic/equilibrium intermediates [13], [45], [46]). Thus, molecular chaperones emerge as an important checkpoint in the folding of PH1 mutants, likely by partitioning AGT folding intermediates into productive formation of native dimers and peroxisomal import, presentation of partially folded states to the mitochondrial import systems, aggregation and proteasomal degradation [7]. Hsp70, Hsp60 and Hsp90 chaperone systems are known to cooperate in assisting protein folding, and the regulation of chaperone activity by cochaperones and regulatory proteins may lead to different fates (i.e. folding vs. degradation) for the client proteins [13]. Overall, all these results suggest that at least these three chaperone systems (Hsp60, Hsp70 and Hsp90) are potential targets for correction of the folding defects displayed by PH1 mutants. Consequently, the detailed characterization of the chaperone requirements for efficient folding of PH1 mutants will open new approaches for therapeutic intervention in PH1. We have already initiated such studies in cell and animal models of PH1 (ongoing work).

## Conclusions

In this work, we present a multidisciplinary approach that provides clues on the protein homeostasis defects displayed by PH1 causing mutations leading to protein aggregation and mistargeting. We observe a significant correlation between mutation-induced kinetic destabilization of the apo-AGT dimer, kinetic trapping by molecular chaperones and intracellular protein foldability and mistargeting. Detailed kinetic and structure-energetics analyses also show that cofactor induced overstabilization of some mutants is caused by subtle changes in the enthalpic/entropic contributions to denaturation free energy barriers, which may also explain the pyridoxine responsiveness found in patients carrying these mutations. We propose that native state kinetic stabilizers and protein homeostasis modulators may be suitable pharmacological therapies to correct folding and stability defects in PH1.

## Supporting Information

Figure S1 **Interaction of Pex5p-pbd and AGT-WT by isothermal titration calorimetry (ITC).** A) Raw calorimetric data; B) Binding isotherm (the line shows the best-fit to one-independent-type-of sites).
(TIF)

Figure S2 **Equilibrium (A) and kinetic (B) PLP binding experiments to apo-AGT.** Line in panel A shows the best fit to a 1∶1 equilibrium binding model; Line in panel B are linear fits of the experimental data, the slope providing the value of *k*_on_ and the y-intercept the value of *k*_off_. Data are from means±s.d. from three independent experiments.
(TIF)

Figure S3 **Exponential relationship between the kinetic stability at physiological temperature and** ***T*** **_m_ values for holo- (open symbols) and apo-(closed symbols) AGT enzymes.**
(TIF)

Table S1 **Thermodynamic binding parameters for the interaction between holo-AGT variants with Pex5p-pbd.**
(DOC)

Table S2 **Data collection and refinement statistics.**
(DOC)

Materials S1 **Materials and Methods.**
(DOC)
