# Supplementary figures and images for: The Role of Protein Denaturation Energetics and Molecular Chaperones in the Aggregation and Mistargeting of Mutants Causing Primary Hyperoxaluria Type I
Source: PLoS One. 2013 Aug 27;8(8):e71963. doi: 10.1371/journal.pone.0071963 (PMC3796444; doi:10.1371/journal.pone.0071963)

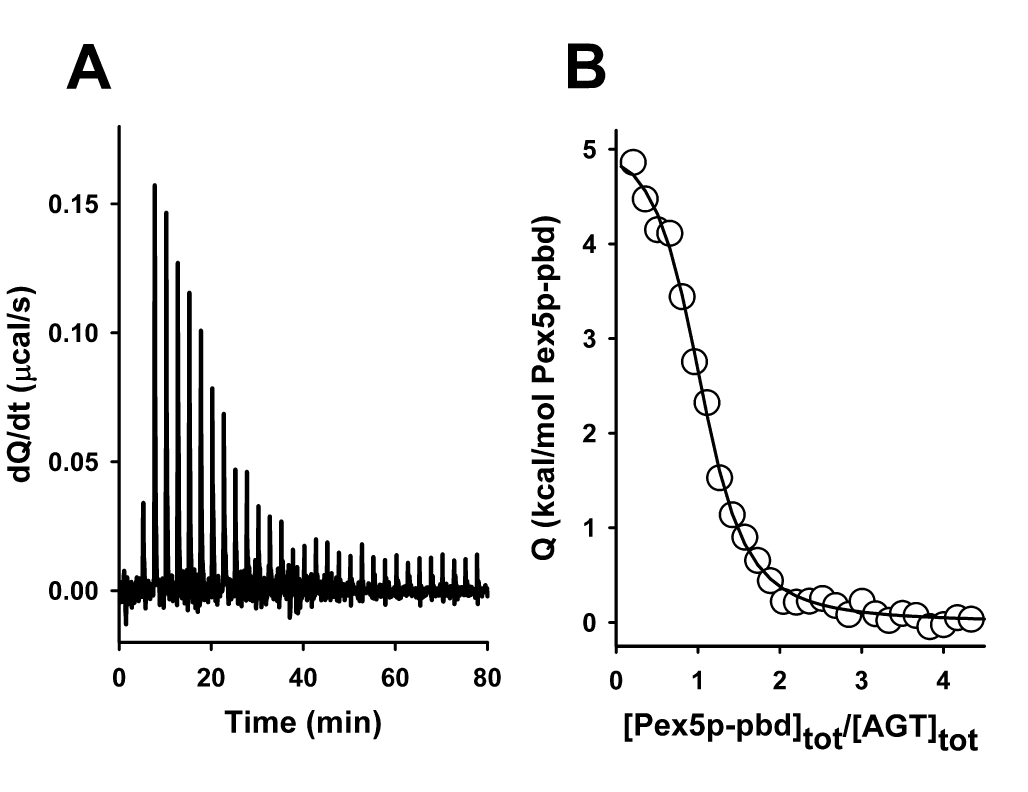

Supplement: Figure S1 — Interaction of Pex5p-pbd and AGT-WT by isothermal titration calorimetry (ITC). A) Raw calorimetric data; B) Binding isotherm (the line shows the best-fit to one-independent-type-of sites). (TIF) [file pone.0071963.s001.tif]

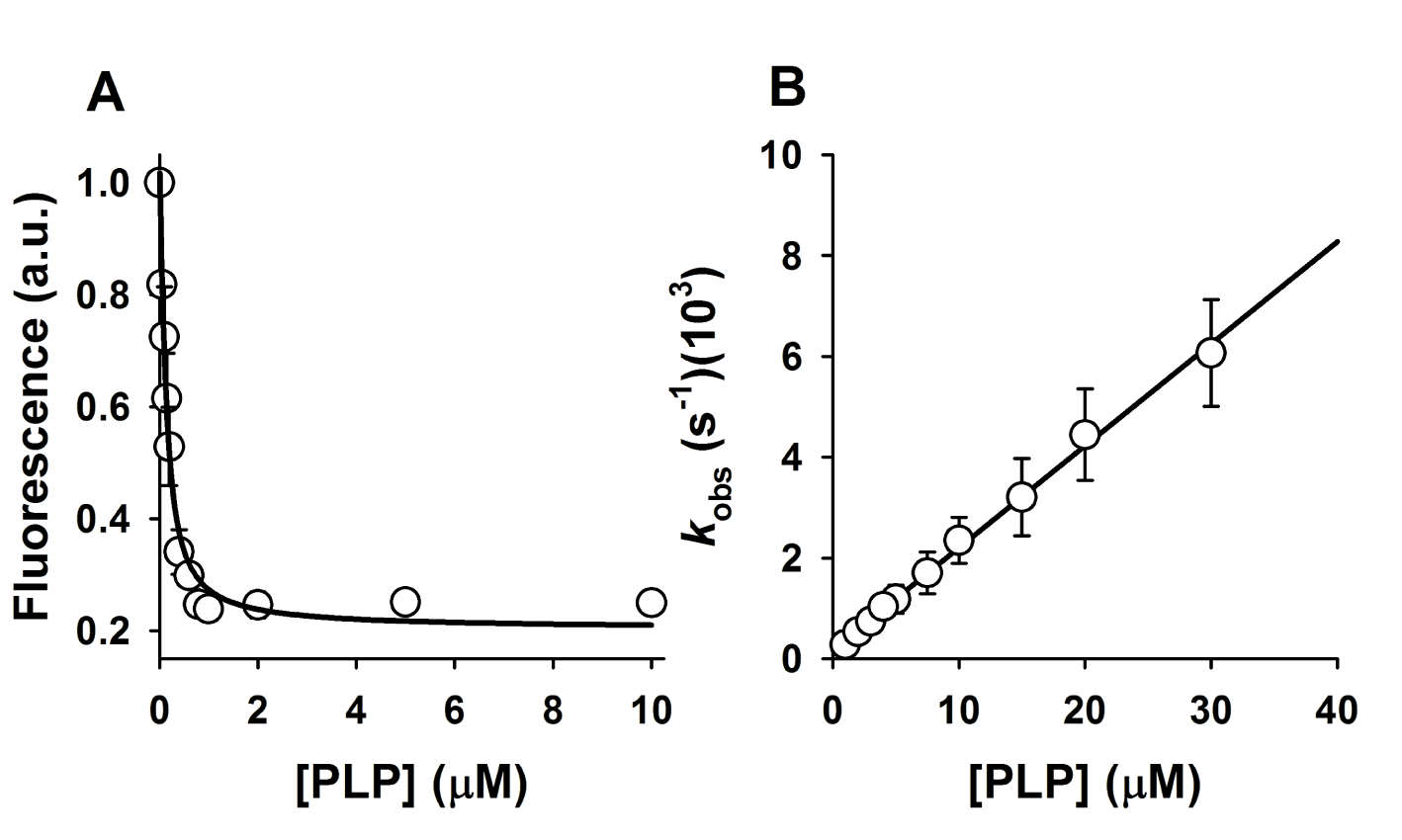

Supplement: Figure S2 — Equilibrium (A) and kinetic (B) PLP binding experiments to apo-AGT. Line in panel A shows the best fit to a 1∶1 equilibrium binding model; Line in panel B are linear fits of the experimental data, the slope providing the value of k on and the y-intercept the value of k off. Data are from means±s.d. from three independent experiments. (TIF) [file pone.0071963.s002.tif]

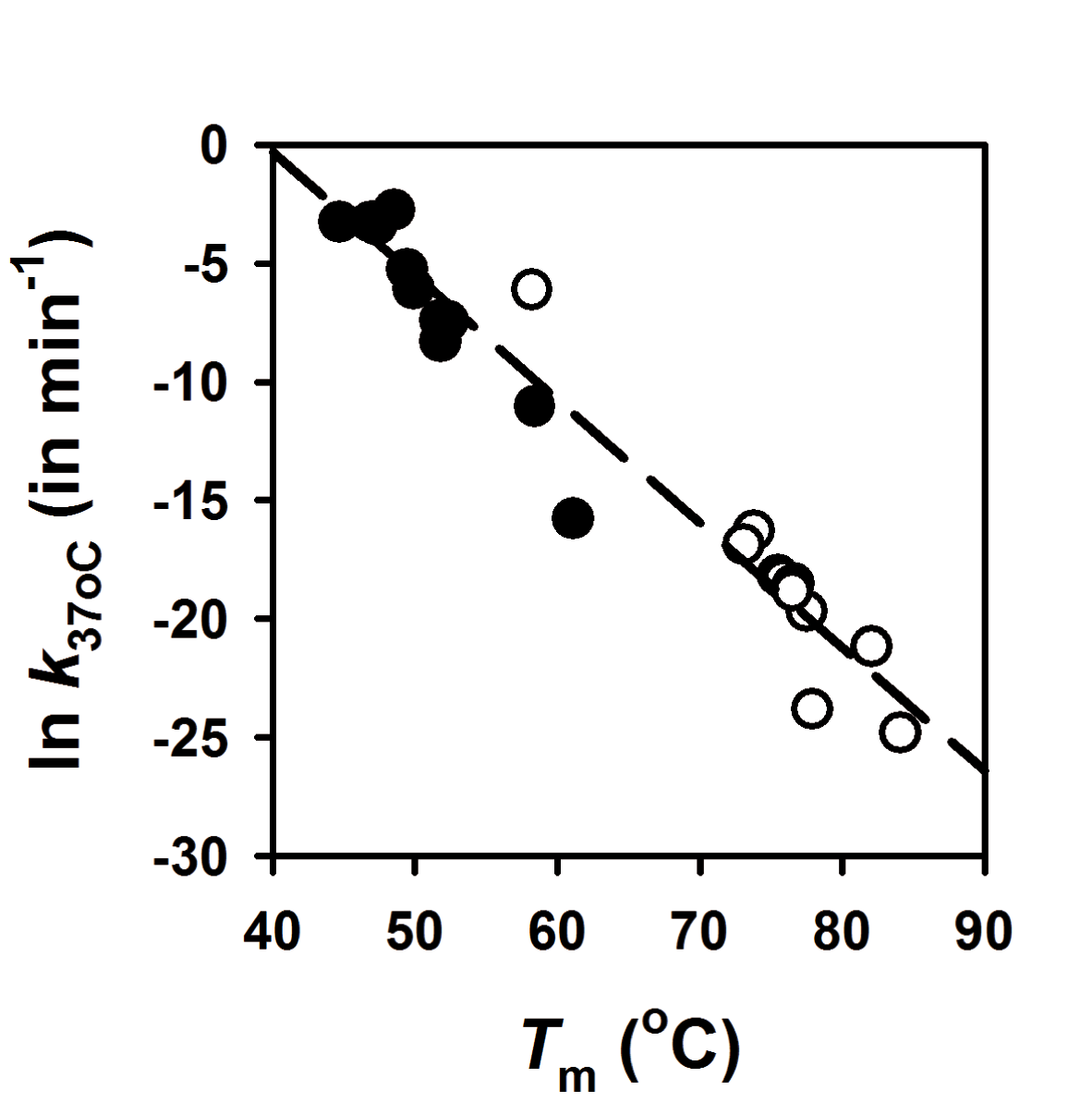

Supplement: Figure S3 — Exponential relationship between the kinetic stability at physiological temperature and T m values for holo- (open symbols) and apo-(closed symbols) AGT enzymes. (TIF) [file pone.0071963.s003.tif]
