# Supplementary material for: The Role of Protein Denaturation Energetics and Molecular Chaperones in the Aggregation and Mistargeting of Mutants Causing Primary Hyperoxaluria Type I
Source: PLoS One. 2013 Aug 27;8(8):e71963. doi: 10.1371/journal.pone.0071963 (PMC3796444; doi:10.1371/journal.pone.0071963)
Supplement: Table S1 — Thermodynamic binding parameters for the interaction between holo-AGT variants with Pex5p-pbd. (DOC) [file pone.0071963.s004.doc]

**Table S1.** Thermodynamic binding parameters for the interaction between holo-AGT variants with Pex5p-pbd.

| **AGT variant** | **N** | ***K*_d_ (µM)** | **Δ*H* (kcal.mol^-1^)** | **-*T*Δ*S* (kcal.mol^-1^)** |
| --- | --- | --- | --- | --- |
| **WT*** | 1.0±0.1 | 1.4±0.2 | 5.2±0.9 | -13.0±0.3 |
| **p.P11L** | 1.05±0.01 | 2.0±0.2 | 5.3±0.1 | -13.1 |
| **p.I340M** | 0.82±0.02 | 1.2±0.2 | 5.0±0.1 | -13.1 |
| **LM** | 0.91±0.02 | 1.1±0.2 | 6.0±0.2 | -14.2 |
| **p.H83R** | 1.12±0.11 | 2.0±0.6 | 5.3±0.7 | -12.5 |
| **p.F152I** | 0.88±0.03 | 2.3±0.4 | 5.9±0.3 | -13.6 |
| **p.G170R*** | 1.1±0.1 | 1.6±0.4 | 5.1±0.1 | -13.2±0.9 |
| **p.R197Q** | 0.87±0.05 | 1.8±0.4 | 5.4±0.4 | -12.9 |
| **p.I244T** | 0.80±0.02 | 1.6±0.2 | 6.0±0.2 | -13.9 |
| **p.A295T** | 0.88±0.05 | 2.2±0.5 | 6.8±0.5 | -14.5 |
| **p.P319L** | 0.93±0.05 | 2.4±0.8 | 5.0±0.5 | -12.7 |
| **p.A368T** | 1.06±0.04 | 1.7±0.3 | 5.7±0.3 | -13.6 |

* Data are mean±s.d. from three independent titrations; otherwise, data are from a single experiment.
