# Supplementary material for: The Role of Protein Denaturation Energetics and Molecular Chaperones in the Aggregation and Mistargeting of Mutants Causing Primary Hyperoxaluria Type I
Source: PLoS One. 2013 Aug 27;8(8):e71963. doi: 10.1371/journal.pone.0071963 (PMC3796444; doi:10.1371/journal.pone.0071963)
Supplement: Table S2 — Data collection and refinement statistics. (DOC) [file pone.0071963.s005.doc]

**Table S2:** Data collection and refinement statistics

| **Crystal data** |  |
| --- | --- |
| Space group | *P* 2_1_2_1_2_1_ |
| Cell dimensions *a*, *b*, *c* (Å) | 54.5 103.5 153.8 |
| *Z* | 2 |
| V_M_ (Å^3^ Da^-1^) | 2.52 |
| Solvent content (%) | 51.28 |
| **Diffraction protocol** |  |
| Radiation source | Synchrotron radiation (ID 14.4) |
| Wavelength (Å) | 0.94 |
| Detector type | ADSC Q4 CCD |
| X ray beam size (microns) | 100 |
| Crystal to detector distance | 297.86 |
| Dp increment per image | 0.5 |
| Temperature (K) | 100 |
| **Data collection statistics** |  |
| Resolution (Å) | 48.22-1.91 (2.0-1.91) |
| No of unique/observed reflections | 69440/603367 |
| Completeness (%) | 99.8 (99.0) |
| Multiplicity | 8.7 (7.7) |
| I / σ(I) | 20.2 (3.2) |
| R_sym_, R_pim_ | 0.07 (0.61), 0.026 (0.23) |
| **Refinement** |  |
| Resolution (Å) | 45.95–1.90 |
| R_work_ / R_free_ | 0.17/ 0.20 |
| No. atoms | 6734 |
| Number of reflections | 69359 |
| Model |  |
| Molecules per a.u. | 2 |
| Aminoacids | 770 |
| Water molecules | 702 |
| Average B-factors | 31.6 |
| R.m.s deviations |  |
| Bond lengths (Å) | 0.007 |
| Bond angles (°) | 1.06 |
| Ramachandran Plot statistics | 98.6 % in the core |
|  | 1.4 % in the allowed |
|  | 0 % outliers |
