# Supplementary material for: The Role of Protein Denaturation Energetics and Molecular Chaperones in the Aggregation and Mistargeting of Mutants Causing Primary Hyperoxaluria Type I
Source: PLoS One. 2013 Aug 27;8(8):e71963. doi: 10.1371/journal.pone.0071963 (PMC3796444; doi:10.1371/journal.pone.0071963)
Supplement: Materials S1 — Materials and Methods. (DOC) [file pone.0071963.s006.doc]

**The role of protein denaturation energetics and molecular chaperones in the aggregation and mistargeting of mutants causing primary hyperoxaluria type I.**

**Noel Mesa-Torres, Israel Fabelo-Rosa, Debora Riverol, Cristina Yunta, Armando Albert, Eduardo Salido and Angel L. Pey.**

**Supplementary Information**

**Material and methods**

Differential scanning calorimetry.-Experimental DSC traces (apparent molar heat capacities vs. Temperature) were fitted using the following expression, which takes into account the experimental chemical baseline:

$C_{p}^{app}=Cp(pre)+(Cp(post)-Cp(pre))\cdot(1-X_{N})-\Delta H\cdot\left(\frac{dX_{N}}{dT}\right)$ (Equation S1)

Where *X*_N_ is the mole fraction of native state, Δ*H* is the denaturation enthalpy and Cp(pre) and Cp(post) are the pre- and post-transition baselines which are considered to be a linear function of temperature. Equation S1 implicitly assumes that only two states are significantly populated. Expressions for X_N_ and dX_N_/dT for the cases relevant in this work are given below.

1. Two-state irreversible model with first-order kinetics

Two-state irreversible models assume that only the native state (N) and the irreversibly denatured protein (the “final” state, F) are significantly populated during the thermal denaturation process and that the conversion from N to F is a purely kinetic process determined by a temperature-dependent rate. All two-state irreversible models can be viewed as limiting cases of more general Lumry-Eyring models corresponding to the situation in which irreversible alteration steps are fast enough to make negligible the population of unfolded and partially-unfolded “intermediate” states.

Assuming that the rate of the overall irreversible process is first-order and, furthermore, that the first-order rate constant changes with temperature according to the Arrhenius equation, the fraction of native protein and its temperature dependence are given by [1,2]:

$$X_{N}=\exp\left[-\exp\left(\frac{E_{a}\Delta T}{RT_{m}^{2}}\right)\right]$$

$$\frac{dX_{N}}{dT}=-\frac{E_{a}}{RT_{m}^{2}}\cdot\exp\left(\frac{E_{a}\Delta T}{RT_{m}^{2}}\right)\cdot\exp\left[-\exp\left(\frac{E_{a}\Delta T}{RT_{m}^{2}}\right)\right]$$

1. Two-state irreversible model with non first-order kinetics

A likely cause of non-first-order kinetics in two-state irreversible denaturation is a difference in oligomerization state between the native protein and the transition state for the rate-limiting step. For instance, if the native protein dissociates in μ “subunits” upon reaching the transition state, the reaction order is 1/μ and the following expressions for *X*_N_ and $\frac{dX_{N}}{dT}$ can be easily shown to hold [2]:

$$X_{N}=\left[1+\frac{(1-\mu)}{\mu}\exp\left(\frac{E_{a}\Delta T}{RT_{m}^{2}}\right)\right]^{(\mu/(\mu-1))}$$

$$\frac{dX_{N}}{dT}=-\frac{E_{a}}{RT_{m}^{2}}\cdot\exp\left(\frac{E_{a}\Delta T}{RT_{m}^{2}}\right)\cdot\left[1+\frac{(1-\mu)}{\mu}\exp\left(\frac{E_{a}\Delta T}{RT_{m}^{2}}\right)\right]^{(1/(\mu-1)}$$

Clearly, when there is no difference in oligomerization state between the native protein and the transition state (for instance, a dimeric native protein and a dimeric transition state), μ=1 and the reaction is first-order. Indeed, taking the μ→1 limit in the two equations above, the ones corresponding to first-order kinetics are obtained [2].

All fittings were performed using MLAB (Civilized Software Inc.).

Fluorescence titrations. For equilibrium titrations, apo-AGT (0.1 µM) was incubated with varying concentrations of PLP (0.05-10 µM) for 4 h at 30^o^C until a stable fluorescence signal was achieved. For p.P11L, p.H83R, p.F152I, p.G170R, p.I244T and p.P319L mutants, time-dependent loss of fluorescence was observed in equilibrium titrations due to protein aggregation. For these mutants, *K*_d_ values were estimated from kinetic experiments under pseudo-first order conditions, using 1-30 µM PLP and 0.1 µM apo-AGT concentrations. Plots of the pseudo-first order rate constants (*k*_obs_) vs. PLP concentration yield the second-order association rate constant (*k*_on_) and the first-order dissociation rate constant (*k*_off_) (Figure S4B), while *K*_d_ values are obtained using *K*_d_=*k*_off_/*k*_on_. The *K*_d_ values obtained by this kinetic procedure are larger than those found by equilibrium experiments (Table 2), owing to the large uncertainty involved in the estimation of *k*_off_. PLP concentration was determined spectrophotometrically using ε_388_ =4900 M^-1^ cm^-1^ [3].

Isothermal titration calorimetry.- Calorimetric titrations were carried out using a MicroCal ITC_200_  microcalorimeter (GE Healthcare), with an operating cell volume of 205.9 μL. All the experiments were performed using 20 mM Na-Hepes 200 mM NaCl pH 7.4. AGT holo-proteins (12-20 μM in protein subunit in the cell) were titrated using Pex5p-pbd (250-350 μM). Each titration was initiated by a 0.5 μL injection followed by 20-30 injections of 1.2-1.7 μL (spaced 150 s). Heats of dilution were determined experimentally in blank titrations and subtracted. The binding stoichiometries (N), dissociation constants (*K*_d_), binding enthalpy (Δ*H*) and entropy (Δ*S*) were obtained by non-linear regression analysis using a one-independent-type-of-sites binding model implemented in the Origin 7.0. software.

**References**

1. Rodriguez-Larrea D, Minning S, Borchert TV, Sanchez-Ruiz JM (2006) Role of solvation barriers in protein kinetic stability. J Mol Biol 360: 715-724.

2. Sanchez-Ruiz JM (1992) Theoretical analysis of Lumry-Eyring models in differential scanning calorimetry. Biophys J 61: 921-935.

3. Peterson EA, Sober HA (1954) Preparation of crystalline phosphorylated derivatives of vitamin B6. JAmChemSoc 76: 169-175.
